# Zika virus replication and cytopathic effects in liver cells

**DOI:** 10.1371/journal.pone.0214016

**Published:** 2019-03-20

**Authors:** Kenneth E. Sherman, Susan D. Rouster, Ling X. Kong, Matthew T. Aliota, Jason T. Blackard, Gary E. Dean

**Affiliations:** 1 University of Cincinnati College of Medicine, Internal Medicine, Cincinnati, Ohio, United States of America; 2 University of Wisconsin-Madison, Madison, Wisconsin, United States of America; 3 University of Cincinnati College of Medicine, Molecular Genetics, Cincinnati, Ohio, United States of America; Saint Louis University, UNITED STATES

## Abstract

Zika virus (ZIKV) has emerged globally as an important pathogen, since it has been recognized as a cause of microcephaly and other neurologic processes and sequalae in newborns. The virus shares homology with Hepaciviruses and therefore may be a cause of hepatitis. We sought to characterize ZIKV replication in hepatocyte-derived cell lines. Huh7.5 and HepG2 cells were infected with ZIKV and replication potential was evaluated by multiple methods including plaque assay, qRT-PCR, negative-strand ZIKV RNA production, and ZIKV NS1 protein production. Growth curves in cells and supernatant were compared to replicative capacity in Vero cells. Overall, viral replication in both hepatocyte lines approximated that observed in the Vero cells. Cell cytopathology was observed after 3 days of infection and apoptosis markers increased. Transmission electron microscopy revealed evidence of viral capsids in cells and negative staining revealed ZIKV particles in the supernatant. *Conclusions*: Hepatocyte-derived cell lines are permissive for ZIKV replication and produce an overt cytopathic effect consistent with development of an acute viral hepatitis. Further evaluation of replication and injury is warranted.

## Introduction

Zika virus (ZIKV) is a single-stranded, positive-sense RNA virus that belongs to the *Flaviviridae* family. There are at least 60 species of flaviviruses classified into four genera, including flavivirus, pestivirus, hepacivirus, and pegivirus. Although first isolated in 1947 in Uganda, ZIKV achieved international prominence in 2016 following a massive outbreak in Brazil that spread through much of South and Central America, and the Caribbean. The international focus on this outbreak was related to its explosive spread which was facilitated by travel and transmission through a mosquito vector and its devastating effects on fetal development in gravid women who were exposed. ZIKV is a mosquito-borne flavivirus and shares structural homology with distantly related hepaciviruses such as hepatitis C virus. [1] Importantly, ZIKV and related flaviviruses are also recognized for their ability to cause hepatitis. Indeed, some of the earliest reports of Zika virus infection were reported in central and western Africa associated with development of jaundice and liver failure.[2] Many flaviviruses can infect hepatocytes and are associated with development of hepatic injury due to direct cytotoxicity or to immune-mediated clearance mechanisms.

Early in the ZIKV epidemic, many laboratories sought to identify cell lines that would support growth and permit study and evaluation of viral strains and to facilitate development of potential vaccine stocks. Though many labs focused on utilization of African Green Monkey Kidney cell lines, several reported ZIKV replication in cultured hepatocytes.[3, 4] We sought to better characterize ZIKV replication and outcomes in hepatocyte cell lines as a prelude to studies of interactions with other viruses that have not been investigated to date.

## Methods

### Zika virus stock

The Zika virus stock isolate was obtained through BEI Resources, NIAID, NIH: Zika Virus, PRVABC59, NR-50240, which was isolated from human blood from Puerto Rico. This isolate is of Asian lineage, introduced in Brazil in 2015 by people traveling from one or more Pacific countries.[5] [6]

### Inoculation into hepatocyte cell lines

ZIKV infection was performed in human hepatocyte cell lines: Huh7.5 derived from human hepatoma cells (provided by Apath LLC, St. Louis, MO), and HepG2 (ATCC HB-8065, derived from human hepatocellular carcinoma cells), as well as Vero C1008 (ATCC CRL-1586, African green monkey kidney cells) which are routinely used in ZIKV research. Cell lines were maintained at 37°C with 5% CO_2_ and cell line-appropriate media. Prior to infection, each cell line was seeded in duplicate into 6-well plates and incubated overnight to reach 70–90% confluency. Viral inoculations were performed at a multiplicity of infection (MOI) rate of 0.27 PFU/cell, and then plates were returned to the incubator for 2 hr to allow virus to adsorb, before the addition of media. This MOI was selected based upon preliminary experiments which indicated that higher MOIs led to such rapid cell loss that replication could not be adequately evaluated. Supernatants and cell monolayers were harvested on days 1, 2, 3, 6, and 8 post-infection. Two additional wells per cell line were harvested at day 3 for electron microscopy processing. In addition, for each cell line, 2 wells of mock infected cells were included.

### M30 apoptosis detection by ELISA

We utilized the M30 CytoDeath ELISA (Diapharma, West Chester, OH) to detect and quantify apoptosis in the cell cultures after exposure to ZIKV over time. This one-step immunoassay measures the accumulation of a caspase-cleaved protein fragment (CK18) released into the culture media after cell death. At each timepoint post-inoculation, aliquots of cell culture supernatant were collected and stored at -20°C until assayed. 25 uL of each sample were tested undiluted in duplicate according to the manufacturer’s instructions. Briefly, standards and samples were added to the antibody-coated 96-well plates, along with HRP conjugate, followed by incubation for 4 hrs at room temperature. After plate washing, 200 uL TMB substrate solution was added and incubated for 20 min at room temperature, in the dark. After the reaction was stopped with sulfuric acid, the absorbance was read at 450 nm and the M30 concentration calculated using a cubic spline fitting algorithm. The assay detection limit is 60 U/L, and the lower limit of quantification is 250 U/L.

### Evaluation of ZIKV replication

Four methods of characterization of ZIKV replication were utilized. These included determination of end-point dilution titer by plaque assay, quantitation using real-time PCR, detection of negative-strand RNA, and quantitation of ZIKV NS1 antigen.

### ZIKV plaque assay

Plaque assays were used to quantify infectious virus produced by the target cell lines over time. Viral supernatants from the target cell lines were serially diluted 10-fold in growth media. Then 0.1 mL aliquots were added to duplicate wells of Vero cells and the virus was allowed to adsorb. After a one hour incubation, the supernatants were removed, and three mLs of an overlay mixture containing 1.2% oxoid agar, 2x DMEM (Gibco, Carlsbad, CA),10% (vol/vol) FBS, and 2% (vol/vol) penicillin/streptomycin was added. Plates were incubated for four days at 37 °C in 5% CO_2_ for plaque development. Cells were then stained with 0.33% neutral red (Gibco) in a 1:1 mixture of 1.2% oxoid agar, 2x DMEM, 2% (vol/vol) FBS, and 2% (vol/vol) penicillin/streptomycin. After an overnight incubation at 37 °C, the plaques were then counted.

### Detection of ZIKV by qRT-PCR

Viral RNA was extracted from 140 uL of target cell line supernatants using the Qiagen QiaAmp Viral RNA minikit, according to the manufacturer’s recommendations. Two uL of extracted RNA was used in a qRT-PCR assay adapted from a method previously described by Xu, *et al*.[7] The primers were designed to amplify a conserved region between the NS5 and 3’ UTR, but were modified to include two degenerate nucleotides in the reverse primer to improve virus detection. (ZIKVF:5’-AGG ATC ATA GGT GAT GAA GAA AAG T-3’ and ZIKVR:5’- CCT GAC AAC AYT AAR ATT GGT GC-3’.) Amplifications were performed in a 20 uL reaction volume, containing Agilent Brilliant III UltraFast SYBR green QRT-PCR master mix, 200 nM of each primer, 100 mM DTT, RT/Rnase block, and the extracted RNA. The qRT-PCR cycling conditions were 50°C for 10 min, 95°C for 3 min, and 40 cycles of 95°C for 5 s, 60°C for 15 s, followed by a melting curve cycle of 65–95°C in 0.4°C/s. All amplifications were performed in triplicate, and each run included all appropriate positive and negative controls.

### ZIKV NS1 ELISA

Zika virus nonstructural (NS1) protein was quantified in cell culture supernatants using a highly sensitive monoclonal antibody-based enzyme-linked immunosorbent assay (ELISA) (Zika NS1 MonoTrace, BioFront Technologies). Briefly, supernatants from Zika-infected cell lines were collected, diluted in 1x cell lysis buffer, and incubated for 30 min at 37°C. The samples were centrifuged at 18,000 x g for 2 min, and the supernatant transferred to a new tube. Serial dilutions of samples (1:8 to 1:320) were performed in duplicate, added to pre-coated plates, and assayed following the ELISA manufacturer’s instructions. Absorbance values were analyzed using a standard curve generated from the supplied recombinant Zika virus NS1 protein. The assay limit of detection is 0.03 ng/mL NS1 protein.

### Detection of negative-strand cellular RNA

Strand specific RT-PCR was performed to verify the presence of the replication intermediate negative-strand ZIKV RNA which is a marker of ongoing viral replication. Cellular viral RNA was extracted from the cell lysates harvested at each timepoint using the Ambion RNAqueous Isolation kit. Briefly, 200 uL of binding buffer was added to each well to lyse the cells, which were then removed to a 0.5 mL tube. After adding ethanol, the lysate was gently vortexed and applied to a spin tube to extract the RNA. After washes, the RNA was eluted, aliquoted, and stored at -80° C prior to RT-PCR. cDNA synthesis was conducted using rTth DNA polymerase (Roche Applied Science, Mannheim, Germany) using the method of Lanford, *et al*. and the primers adapted from Xu.[7, 8] For positive-strand detection, the Xu reverse primer was used, and for negative-strand, the forward primer was used. After cDNA synthesis at 55°C and 60°C each for 30 min, 3 uL of cDNA was used in a 50 uL reaction mix containing both primers at 0.2 uM, and Qiagen Taq PCR Master Mix. RT-PCR cycling conditions were 94°C for 1 min, followed by 40 cycles of 94°C for 30 s, 58°C for 1 min, and 72°C for 1 min, and a final extension of 72°C for 10 min. The 113 bp PCR products were observed by agarose gel electrophoresis.

### Electron microscopy

On day 3 of infection, 2 wells per cell line were washed with PBS, flooded with 2.5% glutaraldehyde/cacodylate buffer fixative, and allowed to fix for 10 min at room temperature. The cells were transferred to a centrifuge tube and spun at 320 x g for 10 min to pellet. The fixative supernatant was removed, replaced with fresh fixative, and allowed to fix overnight. The fixative was then removed and replaced with 8% sucrose in 0.1M Sorensen’s PB (Electron Microscopy Sciences, Inc.), allowed to sit for 15 min, and then repeated 2x. The cell pellets were then processed for EM microscopy by the Electron Microscopy Pathology Research Core at Cincinnati Children’s Hospital Medical Center. Images were acquired using a Transmission Electron Microscope (TEM) (JEOL JEM-1230) located in the University of Cincinnati College of Medicine, Department of Cancer Biology. In addition, a negative contrast technique was utilized to help distinguish virus. For this method, 5 uL of virus suspension was incubated with 2.5% glutaraldehyde for 60 min, and then 6 uL of the fixed suspension was dropped onto a BSA pre-coated EM copper grid. After adherence of the virus to the grid, the excess liquid was wicked away, followed by negative staining with 5% uranyl acetate. The grids were allowed to dry, and then viewed on the TEM.

### Statistical analysis

Parametric and non-parametric comparisons were performed as appropriate to the data using Statistix 10.0. (Analytical Software, Tallahassee, FL). A p-value of 0.05 was considered significant. An unpaired Student’s t-test was used to determine significant differences in virus titers. Data are presented as means ± SEM. Comparisons of viral titers were performed by using a repeated measures two-way ANOVA (GraphPad Prism) with Bonferroni post-tests at each time point.

## Results

### Replication in tissue culture

**Fig 1:**
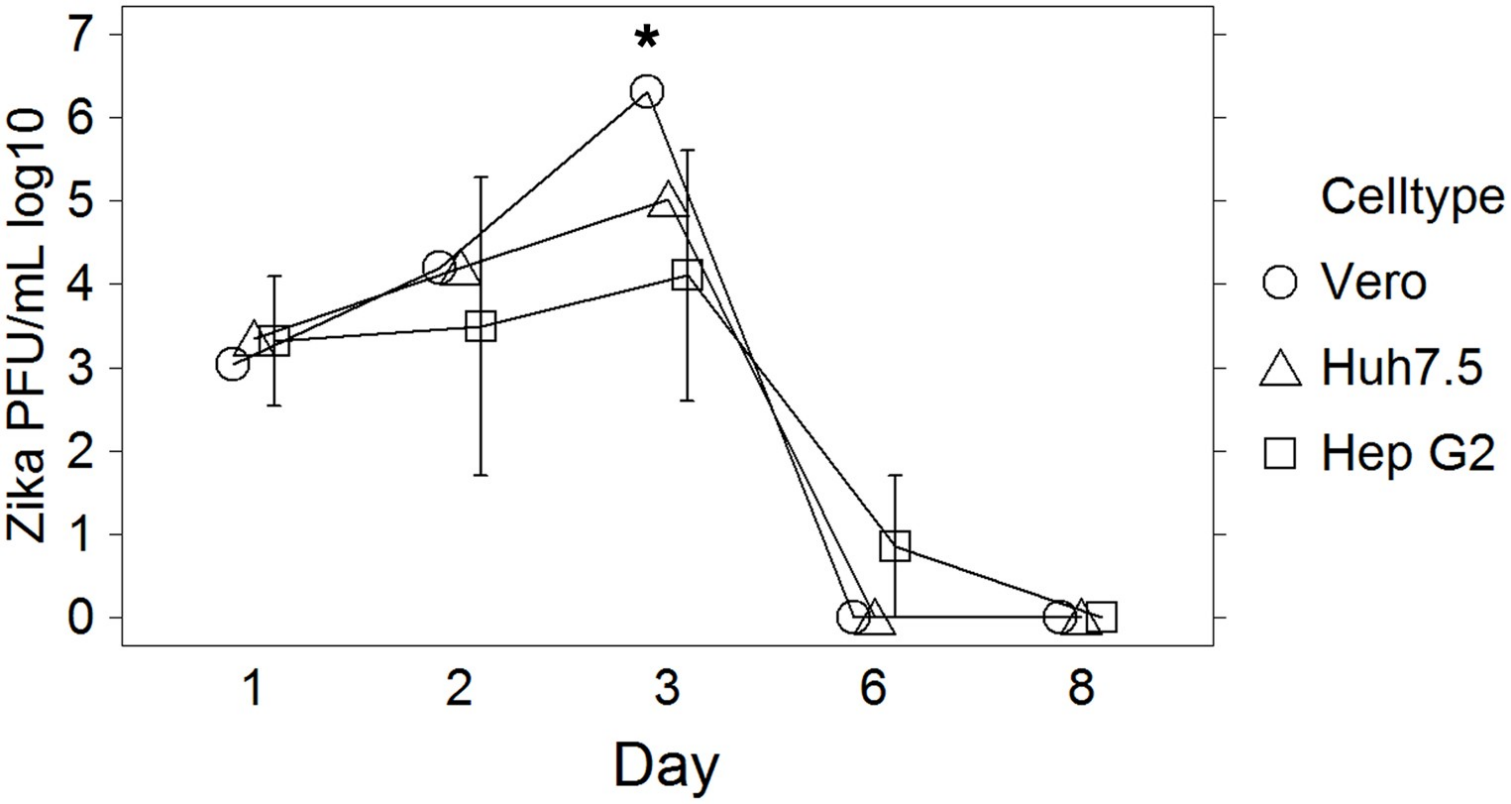
Production of infectious Zika viral particles. In target cell lines over time as measured by plaque assay. PFU/mL = plaque forming units/milliliter. Error rate for plaque assay is +/- 0.5 log. Asterisks denote data with statistically significant change from baseline.

To more fully characterize ZIKV infection of hepatocyte cell lines, four approaches were utilized to determine ZIKV replication competence in these cell lines. First, viral infectivity and replication was assessed *in vitro* using Huh7.5, HepG2, and Vero cells. Viral growth curves were similar between Huh7.5 and HepG2 cells, peaking at day 3.ZIKV appeared to replicate to higher peak titers by day 3 in Vero cells as compared to hepatocyte cell lines. (Fig 1 and S1 Dataset) After day 3 post inoculation (pi), there was a precipitous drop in titer and by day 6 pi the titer was below the plaque assay limit of detection. This was coincident with the development of significant cytopathology that included loss of attachment and cytolysis.

**Fig 2:**
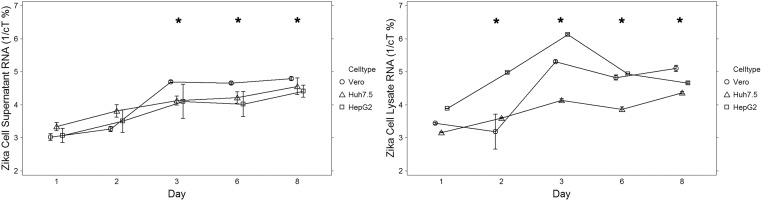
Presence of Zika RNA. Determined by qRT-PCR in target cell lines: Vero, Huh7.5, and HepG2, in a) cell culture supernatants, and b) cell lysates. Results are the percent inverse of the PCR cycles over time of infection. Data are means with bars representing standard error of the mean. Asterisks denote data with statistically significant change from baseline.

Consistent with infectious titer data, ZIKV RNA levels increased significantly between days 1 and 3 in all cell lines, with similar profiles in both supernatant and cell lysates. Supernatant RNA from Huh7.5 cells and HepG2 cells (Fig 2a and S1 Dataset) both demonstrated significant (p<0.05) increases in relative titer between Day 1 and 3, compared to baseline. A plateau was observed between Day 3 and 6, which was also evident in the Vero cell line. During this period, the cells were observed to demonstrate significant injury with loss of attachment and cytolysis. Slightly higher levels were noted on Day 8. Fig 2b illustrates detection of Zika RNA present in the cell lysates.

**Fig 3:**
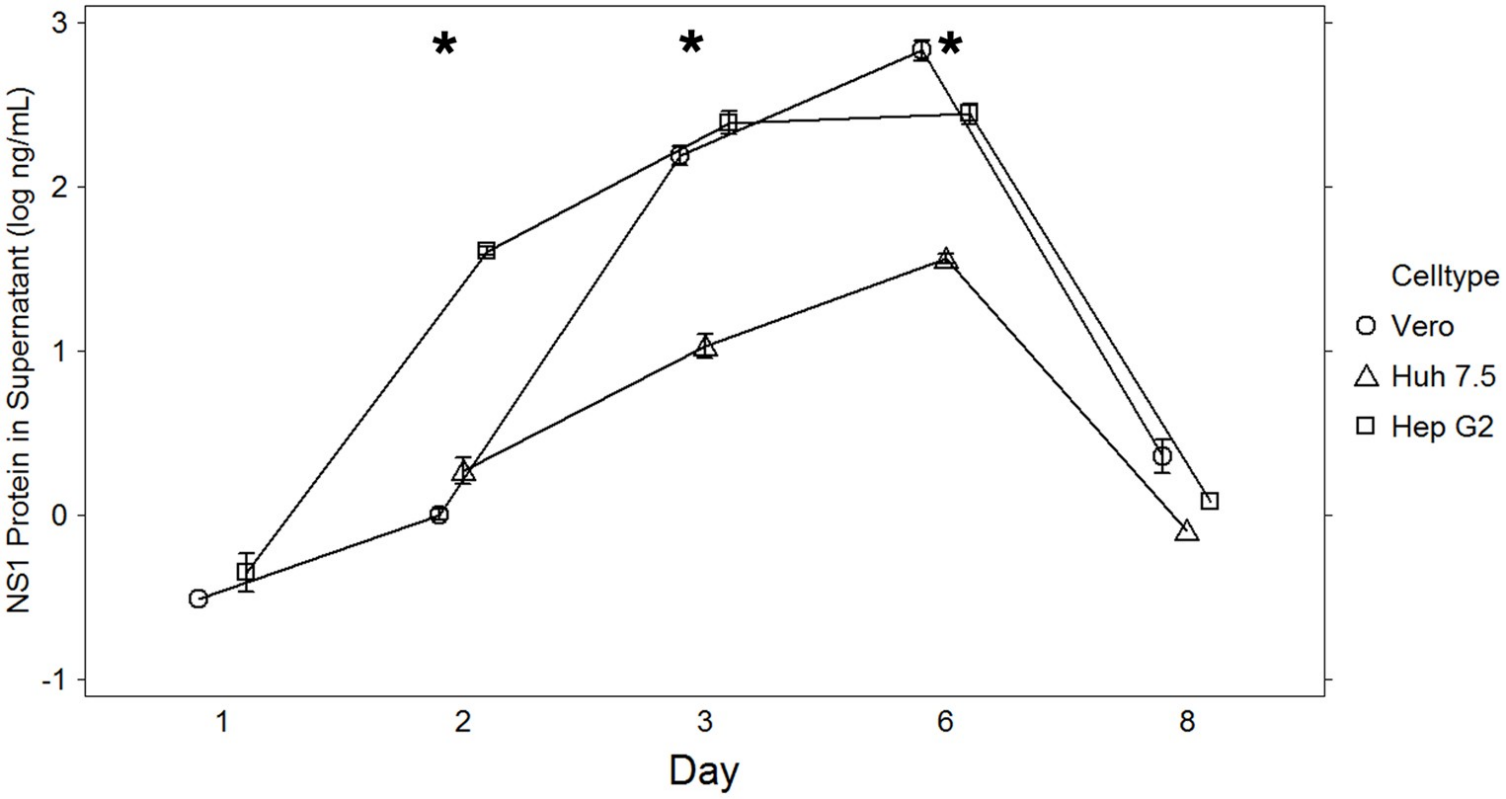
Zika NS1 protein. Measurement of Zika NS1 protein in target cell line supernatant over time, by ELISA. Results are expressed as the log ng/mL of the mean of 2–4 replicates in two experiments. Bars represent standard error of the mean. Asterisks denote data with statistically significant change from baseline.

Next, the production of ZIKV NS1 protein released into the supernatant was examined (Fig 3 and S1 Dataset). Interestingly, in contrast to QRT-PCR and plaque assays, there was delayed ZIKV NS1 protein secretion until after Day 2 in all of the cell lines, with Vero cells showing the highest level of NS1 protein productivity that peaked at Day 6. More modest protein was produced in the HepG2 cells which peaked at Day 6. In Huh 7.5 cells, a nearly 40 fold increase in NS1 was observed but this was less than that seen in the other cell lines. By Day 8 NS1 protein was not detectable in any cell supernatant consistent with degradation of the intact viral particles.

**Fig 4:**
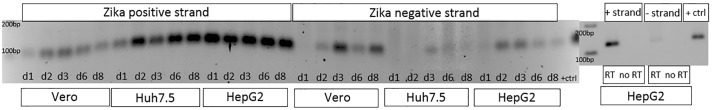
Agarose gel visualization of RT-PCR 113 bp products. Amplification of Zika RNA extracted from cell lysates from the target cell lines, at days 1,2,3,6, and 8 of infection. Positive strand products were amplified using the negative primer and negative strand products using the positive primer in the initial reverse transcription (RT) step. Product size is indicated by a 100 base pair ladder. To verify that no products were present without the initial RT step, representative HepG2 cell lysates were amplified with and without the reverse transcription enzyme. No bands appear in the lanes which had no RT enzyme. Positive strand virus with RT is more abundant than negative strand virus.

We also performed strand specific RT-PCR to verify the presence of negative-strand ZIKV cellular RNA which is considered to be a marker of ongoing viral replication. Replication of the Zika genome occurs via negative-strand intermediate. The rTth DNA polymerase-based strand specific RT-PCR assay in cellular lysates detected the presence of negative-strand Zika RNA at a lower concentration than the positive-strand, as shown in Fig 4. As a negative control, no PCR products were amplified in the no-RT reactions.

### Transmission electron microscopy

**Fig 5:**
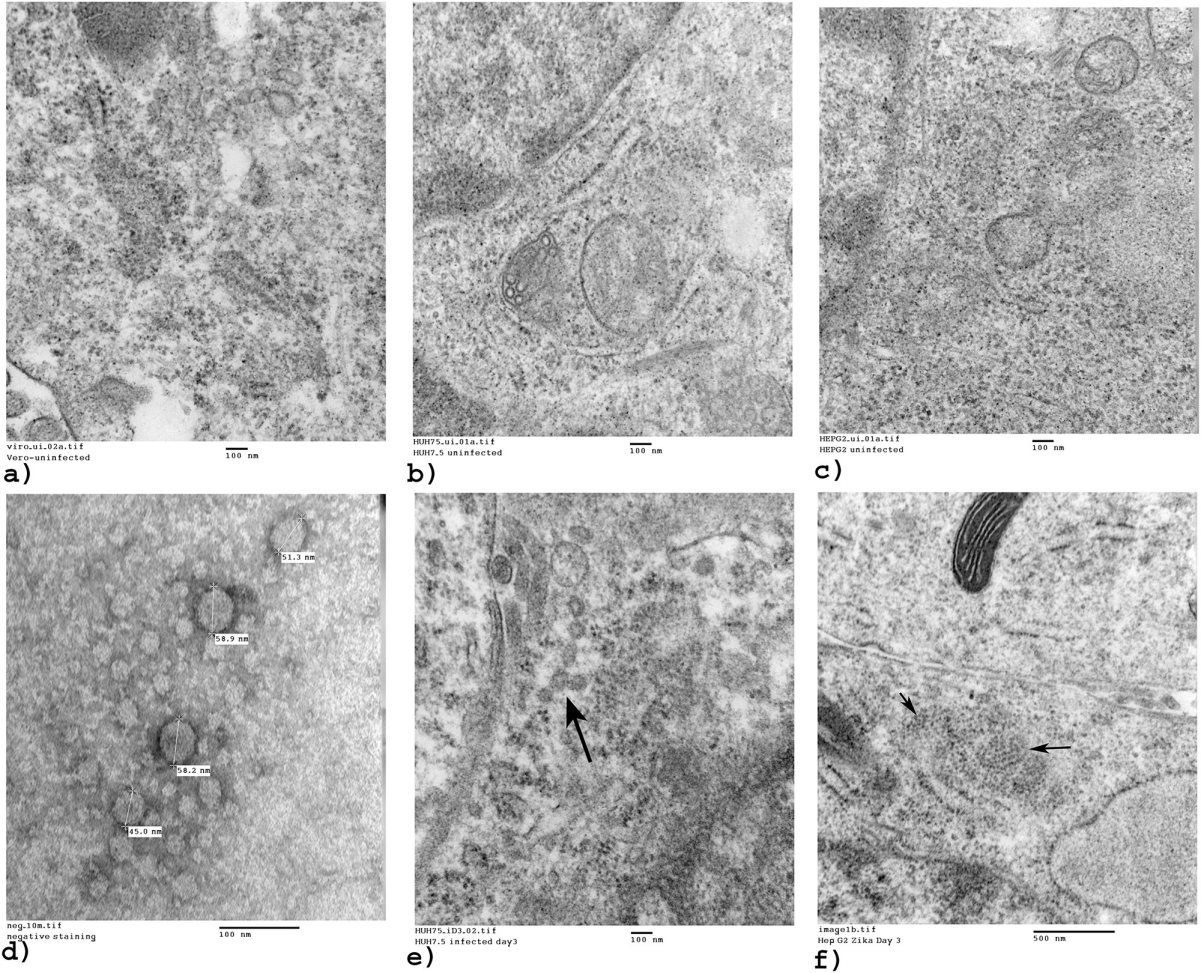
TEM images. Cell lines with either uninfected or Zika-infected cells were visualized by electron microscopy to observe ultrastructure, after three days of infection. Black arrows point to ZIKV. a) EM image of uninfected Vero cells. Magnification = 80000x. Scale bar = 100 nm. b) Uninfected Huh7.5 cells. Magnification = 80000x. Scale bar = 100 nm. c) Uninfected HepG2 cells. Magnification = 80000x. Scale bar = 100 nm. d) EM image using negative staining of Zika virions in Vero cell culture supernatant. Particle sizes in nm. Magnification = 300000x. Scale bar = 100 nm. e) EM image of Zika infection in Huh7.5 cells. Magnification = 80000x. Scale bar = 100 nm. f) EM image of HepG2 cells infected with Zika virus. Magnification = 60000x. Scale bar = 100 nm.

ZIKV was visualized in representative cells using transmission electron microscopy. Uninfected cell lines are shown in Fig 5a, uninfected Vero cells; 5b, uninfected Huh7.5 human hepatoma cells; and 5c), uninfected HepG2, human hepatocellular carcinoma cells. EM images of Zika-infected cell lines illustrate cellular ultrastructure and presence of virions. The image in Fig 5d captures negative staining of virus produced and shed into the supernatant of Vero cell culture. Negative staining with uranyl acetate is a common method used to provide contrast when viewing viruses. Particles ranged in size from 45 to 59 nm. Fig 5e shows ZIKV virus-induced vesicles 3 days after infection of Huh7.5 cells, and virions in HepG2 cells (Fig 5f).

### M30 apoptosis detection by ELISA

**Fig 6:**
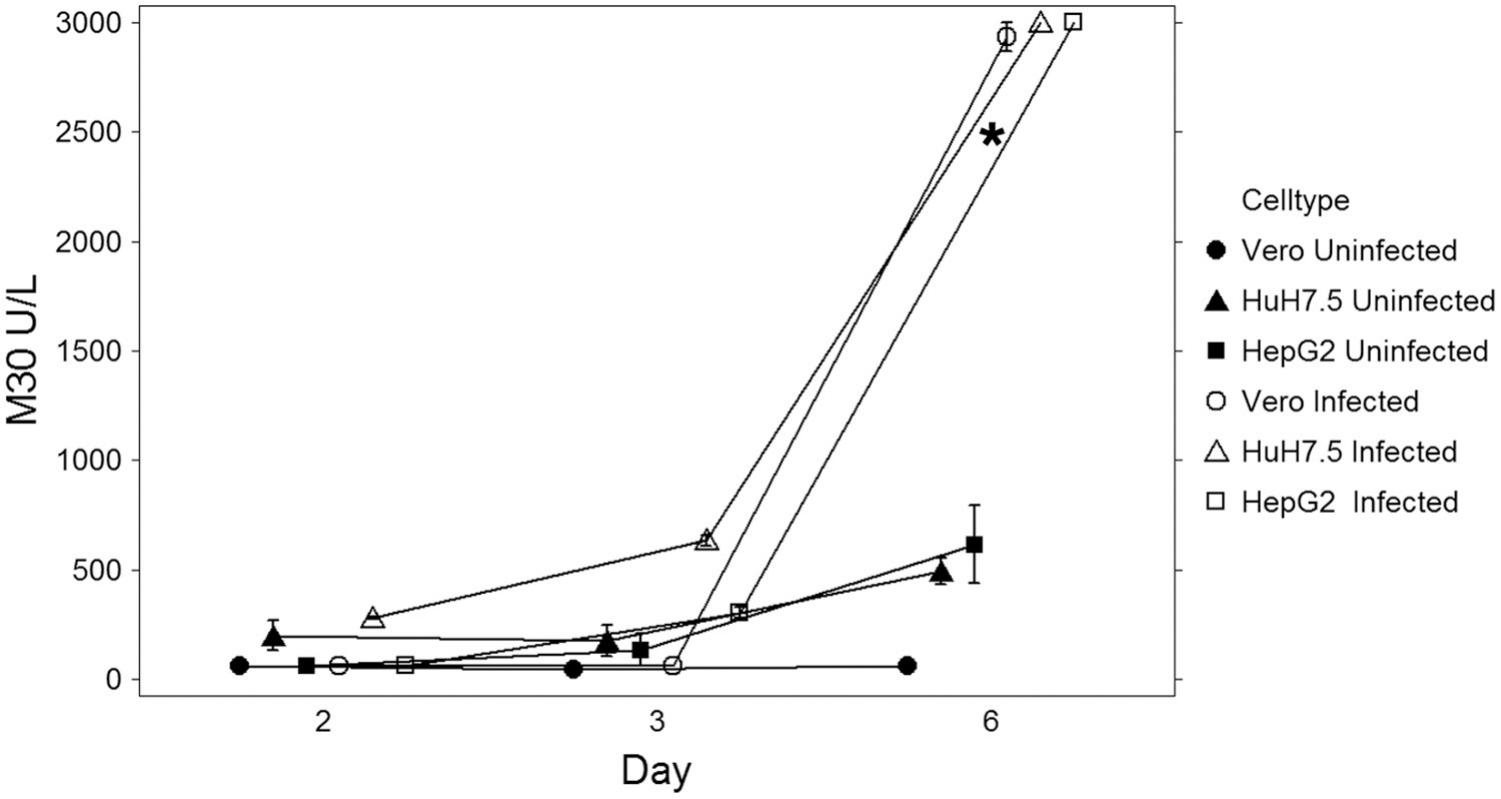
Apoptosis as measured by M30 ELISA. Determination of apoptosis in undiluted cell supernatants over time by M30 CytoDeath ELISA (enzyme-linked immunosorbent assay). Results are the mean of 2–4 replicates, bars representing standard error of the mean. Assay detection limit is 60–3000 U/L; LLOQ is 250 U/L. Asterisk denotes data with statistically significant changes from baseline.

We utilized the M30 CytoDeath ELISA to detect and quantify the M30 antibody (detecting the Asp396 caspase-cleavage site on CK18) as a marker of induced apoptotic cell death. Proteolytic cleavage of CK18 takes place before cellular membrane disruption and this soluble analyte is then released into cell culture supernatants. Fig 6 illustrates M30 determination in uninfected and infected hepatocytes and Vero control cell lines, over up to 6 days post-infection (pi), in undiluted cell culture supernatants. Apoptosis was detected at 3 days post-infection in infected hepatocytes, and CK-18 levels greater than the undiluted assay range of 3000 U/L were observed. These levels were significantly higher than those seen in the uninfected cell lines using adjusted pairwise comparisons (p < 0.001, S 1). Vero cells began to demonstrate signs of apoptosis after day 3 to levels less than those seen in the hepatocyte cell lines, illustrating the susceptibility of liver cells to Zika infection.

## Discussion

In the last two years, ZIKV burst into the attention of both the public and health professionals. Though first isolated from sentinel rhesus living in the Zika forest near Entebbe, Uganda in 1947, it was not described in the literature until 1952 by Dick and colleagues.[9, 10] Initial studies of passage of the isolate in mice suggested that no organ including liver was infected outside of the brain. However, two years later Macnamara described “an epidemic of jaundice” in Nigeria and reported isolation of ZIKV as the implicated causative agent.[2] Yellow fever and other etiologies were excluded within the limits of technology available at that time. ZIKV publications were scarce in the medical literature after the initial reports. In 1979, Fagbami noted that ZIKV induced disease was common, mild and frequently missed in Nigeria.[11] A report describing liver pathology in patients with yellow fever, described histopathologic lesions in patients that were more typical of other forms of viral hepatitis as opposed to yellow fever, and noted that some of these patients had high titers of antibody to ZIKV.[12] In 2013 a new strain of ZIKV (“New World Zika Virus”), probably transferred from Yap in Micronesia which reported a ZIKV outbreak in 2009 rapidly spread through South and Central America and the Caribbean basin.[13] It was first reported in Puerto Rico in 2015 and on the U.S. mainland in 2016.[14, 15] The virus was noted to cause birth defects (including but not limited to microcephaly) in children of mothers infected during pregnancy, a finding which had not been reported previously.

In this report, we describe the replication of ZIKV in hepatocyte cell lines. Liver cell lines appear to be permissive of infection with the strain of ZIKV currently circulating in the Americas. This is supported by multiple lines of evidence including increased ZIKV RNA detected in supernatant after virus exposure by both qRT-PCR and by nested PCR methods. Negative-strand RNA intermediates can also be detected. ZIKV antigen levels rise in supernatant following infection. The virus that is produced can be titered using a biologic assay (plaque assays) which demonstrates increases in infectious virus following culture on hepatocytes. Finally, ZIKV-like particles can be visualized in the supernatant of infected cells and within infected cells. In the search for permissive cell lines, others have noted replication in liver-derived cell lines as well. For instance, Weger-Lucarelli and colleagues noted peak viral titers were observed in Huh7 cells 3–4 days after inoculation using both a parental ZIKV strain and an infectious clone-derived virus.[16] The kinetics of replication was similar between Huh7 and Vero cells when ZIKV RNA was measured. Huh7 cells were also utilized to propagate virus by Vicenti et al.[3] Both plaque assays and qRT-PCR were employed to measure viral production. Virus titers measured by plaque assay peaked at day 3 following infection and declined thereafter. Our observations suggest that significant cytotoxicity due to viral infection is observed in liver cells three days following infection and that cell loss limits virus production. This pattern of cytotoxicity is consistent with that seen in viral hepatitis. However, there has been little focus in clinical literature on liver cell injury related to ZIKV infections. A recent case report described a 29 year-old man infected with ZIKV who developed ALT abnormalities which peaked 10 days after development of fever at 329 U/L. Coagulation abnormalities were also noted. ZIKV was isolated from this patient and passaged in a mosquito-cell line.[17]

In summary, we present an evaluation of ZIKV replication in hepatocyte-derived cell lines, including evidence of the presence of replicative intermediates and electron microscopy demonstrating presence of ZIKV virions in the supernatant. While other investigators have evaluated permissiveness of ZIKV in hepatocytes, their focus was to find cell lines that might support viral growth with the purpose of developing stocks of virus that can be used for vaccine development and other types of studies. Our interest is in the characterization of viral replication as it relates to liver cell injury and to ultimately determine if a persistent state of ZIKV replication might exist in those with an immunocompromised state. To this end, we recently reported that ZIKV infection is relatively common in HIV-infected persons in Ghana, and we will seek to further describe these interactions in places where coinfection might exist.[18] We will also explore specific mechanisms of hepatocyte injury due to Zika infection in subsequent work.

## Supporting information

S1 Dataset Zika data for graphs. (XLSX)
